# China’s New Cooperative Medical Scheme’s Impact on the Medical Expenses of Elderly Rural Migrants

**DOI:** 10.3390/ijerph16244953

**Published:** 2019-12-06

**Authors:** Jiajing Li, Yanran Huang, Stephen Nicholas, Jian Wang

**Affiliations:** 1 Center for Health Economics Experiment and Public Policy, School of Public Health Shandong University, No. 44 Wenhuaxi Road, Lixia District, Jinan 250012, China; sdu_hn@163.com (J.L.); 201715522@mail.sdu.edu.cn (Y.H.); 2 School of Economics and School of Management, Tianjin Normal University, No. 339 Binshui West Avenue, Tianjin 300387, China; stephen.nicholas@newcastle.edu.au; 3 Guangdong Institute for International Strategies, Guangdong University of Foreign Studies, 2 Baiyun North Avenue, Guangzhou 510420, Guangdong, China; 4 Top Education Institute, 1 Central Avenue, Australian Technology Park, Eveleigh, Sydney, NSW 2015, Australia; 5 Newcastle Business School, University of Newcastle, University Drive, Newcastle, NSW 2308, Australia; 6 Dong Fureng Institute of Economic and Social Development, Wuhan University, No. 54 Lishi Lane, Dongcheng District, Beijing 100010, China

**Keywords:** migrants, elderly, the New Cooperative Medical Scheme, medical expenditure, urbanization

## Abstract

Background: With rapid urbanization in China, the scale of elderly migrants from rural areas to urban cities has increased rapidly from 5.03 million in 2000 to 13.4 million people in 2015. Methods: Based on the unbalanced panel data obtained from the Chinese Longitudinal Healthy Longevity Survey, this study investigates the impact of changes to the New Cooperative Medical Scheme (NCMS) on the medical expenditure of Chinese elderly rural migrants by using seemingly unrelated regression models. Results: NCMS coverage for elderly rural migrants rose from 11.83% in 2005 to 87.33% in 2014. The effective reimbursement rate increased significantly from 4.53% in 2005 to 36.44% in 2014, and out-of-pocket/income fell by 50% between 2005 and 2014. The NCMS significantly increased the effective reimbursement rate by 12.4% and out-of-pocket medical expenditure/income by 7.5% during this decade but played an insignificant role in reducing out-of-pocket payments. Conclusions: Policy makers need to promote a two-pronged strategy, which involves controlling the excessive growth of urban medical expenses and continuing to reform NCMS reimbursements for medical treatment, so non-urban resident elderly rural migrants can fully enjoy the welfare benefits of migration and urbanization.

## 1. Introduction

Rapid urbanization has seen the growth of Chinese megacities fed by rural-to-urban migration [1]. In 1980, only 19.6% of the population in China lived in urban areas but by 2011, the urban population surpassed the rural population. Such hypermigration creates vulnerable populations, including unemployed migrants, young out-of-school migrants, migrant sex workers, illegal child migrant laborers and elderly migrants [2]. While rural-to-urban migration was dominated by men in the 1980s and 1990s, by 2005, roughly 35% of rural migrants were women and 21% migrated with their families [3]. The number of elderly migrants—those over 65 years old—also increased rapidly. In 2000, there were 5.03 million elderly rural migrants, accounting for 0.4% of the national population and 4.9% of the national floating population, rising to 13.4 million rural migrants and making up 0.95% of the national population and 5.3% of the national floating population in 2015, with an average annual growth rate of 6.6% [4]. Elderly rural migrants were likely to be less productive and less healthy than non-migrant older people, but little research attention has focused on the social and health needs of older migrant populations in developing countries [5]. Data from China’s Three Gorges Reservoir area suggest that the overall level of physical and psychological health of elderly migrants was lower than that of non-migrant older people [6]. With poorer health outcomes than the rest of the population, elderly migrants are likely to face rapidly increasing medical expenses, with higher medical debts, than non-migrant older people, imposing a significant financial burden on migrant families and China’s national health system [7].

In 2003, the New Cooperative Medical System (NCMS) was launched by the Chinese government, which aimed to open basic health services to the elderly in order to attenuate the burden on rural residents of inpatient and outpatient medical expenses [8]. China’s basic national health insurance system is related to residents’ county-based household registration, or hukou. The NCMS covered rural residents while employed urban residents were insured by the Urban Employee Basic Medical Insurance (UEBMI) and urban residents without formal employment and students were insured by the Urban Resident Basic Medical Insurance (URBMI). The migrant population had limited access to non-hukou the UEBMI or the UEBMI schemes or medical services in urban areas [9], relying on their rural NCMS or self-funded their medical treatment [1]. Since the funding pool of the NCMS was based on the rural county’s economic level, NCMS compensation tied to a resident’s hukou did not cover cross-county medical treatment expenses, especially in urban counties, where health services were more expensive than those in the rural hukou county [10]. While the three basic health insurance schemes covered 95% of the total population, less than 10% of rural migrants enjoyed non-NCMS insurance [11]. In a survey of 752 elderly rural migrants in Shanghai, the participation rate in the urban medical insurance schemes was only 1.9% [12].

Critical features of the NCMS was its “non-carrying” provision and reimbursements. First, the NCMS is not easily portable across counties within most provinces [1], with reimbursements usually claimed in the county where a person is registered [13]. Second, compared with the urban registered UEBMI and URBMI reimbursement rates, the starting line of reimbursements and the nominal reimbursement ratio for the NCMS was lower than that for the UEBMI and the URBMI. Further, the reimbursement process for NCMS rural migrants living in urban places was tedious and time consuming [14], which was an additional barrier to elderly migrants making use of urban medical services [13]. A survey in Shanghai found that the challenges of the medical insurance reimbursement system in urban places meant many elderly rural migrants did not voluntarily see a doctor after falling ill, but choose no medical services or self-treatment [15]. Not only does the NCMS system constrain access to urban medical services, limiting the urbanization dividend for elderly rural migrants, but inhibits elderly migrants’ willingness to migrate. It has been estimated that the NCMS locks-in between 35% and 66% of potential elderly rural migrants to their rural registration county [14].

Issues with the NCMS, including its compensation ability and portability, have long been recognized. With the 2009 health reforms, various adjustments aimed to mitigate the burden of urban health expenditure for rural migrants, promote almost universal health coverage for rural residents, support the growth of personal insurance premiums and expand the reimbursement scope of NCMS insurance. To cover these changes, the annual NCMS premium per capita gradually increased from ¥40 (US$4.88) in 2005 to ¥400 (US$65.11) in 2014. In 2005, the NCMS only covered 21% of the population in China, rising to 98.9% in 2014, comprising 736 million rural people. The insurance coverage mainly involved basic medical services and medicines with a nominal reimbursement ratio for hospital expenses of approximately 30%. As part of the 2009 health reforms, the NCMS was expanded to cover outpatient expenditure. The compensation upper bound was raised, with the clinic observation rate pegged at ¥1000 (US$162.79) and the total medical services expenses limit expanded to ¥200,000 (US$32,557.38). In 2014, the nominal reimbursement ratio ruled by the NCMS for inpatients ranged from 50% to 85% among provincial hospitals to township hospitals [16]. These reforms improved insurance financing and reimbursement compensation of NCMS members.

Empirical studies and a systematic review of the NCMS [8,13,17,18,19,20] reveal that NCMS policies to alleviate the direct medical financial burden on farmers remains a controversial issue. There is little research on NCMS financial protection of the medical expenses of rural migrants. Wen Chen [10] found that the NCMS effectively reduced the proportion of out-of-pocket medical expenses in the total family expenditure for rural and urban migrants aged 15–59, but did not study the impact of the NCMS on elderly migrants. Jing Zhao et al. [21] found that participation in Shanghai’s health insurance increased the likelihood of the intentions for migrants aged 15–64 staying in Shanghai rather than returning to their hometown in their old age. Bo-li Peng et al. [22] found that rural-to-urban migrants aged 15–59 with the NCMS were more likely to return to their former residence for medical treatment, and less likely to live permanently in the relocation area. Han, Junqiang et al. [23] found that the difference between the place of insurance and the place of residence resulted in the inequity in health service utilization by the floating elderly population, but they did not analyze the medical cost. What was the impact of the 2009 NCMS reforms on the health expenditure of elderly rural migrants? By analyzing medical expenses, our study provides an assessment of NCMS policy reforms, including the impact of out-of-pocket (OOP) expenses and reimbursement rates on elderly rural migrants.

## 2. Materials and Methods

### 2.1. Materials

Based on the micro data from the Chinese Longitudinal Healthy Longevity Survey (CLHLS) [24], our focus is on the impact of the NCMS on the direct medical financial burden of rural elderly migrants. CLHLS data source can be found at https://www.icpsr.umich.edu/icpsrweb/DSDR/studies/36179. All subjects gave their informed consent for inclusion before they participated in the study. The study was conducted in accordance with the Declaration of Helsinki, and the protocol was approved by research ethics committees of Duke University and Peking University (IRB00001052–13074).

We used four waves of unbalanced data (2005, 2008, 2011 and 2014) from this survey, which is a large-scale, multidisciplinary, longitudinal follow-up survey of the elderly covered individuals aged 65 and above across 22 Chinese provinces and provincial cities [25,26]. The baseline survey started in 1998 and follow-up surveys of CLHLS were conducted in 2000, 2002, 2005, 2008, 2011 and 2014. Medical insurance coverage and health care expenditure-related questions were asked until the 2005 wave [16]. Therefore, we sampled migrants over 65 years old from rural areas now living in cities from 2005 and subsequent waves (2008, 2011 and 2014) for a comparative China-wide analysis. The sample size was 8658 in the 2005 wave; 10,198 in the 2008 wave; 5153 in the 2011 wave; and 3967 in the 2014 wave.

### 2.2. Variables

Medical expenses comprised all medical expenditures, including the costs of outpatient treatment, pharmacological agents, and hospitalization bills and OOP medical payments refer to all categories of medical expenses paid directly by the household and not reimbursed by health insurance [27]. Medical expenses were obtained from CLHLS and OOP expenditure data from the CLHLS item ‘Medical expenses paid by the family last year out of total medical costs’. Medical insurance premiums were excluded since our focus is on health expenditure directly due to illness. Medical expenses, OOP expenses and per capita income were multiplied by a 8% per year inflation factor [28]. The US$/RMB exchange rates used where 8.192 in 2005, 6.945 in 2008, 6.459 in 2011 and 6.143 in 2014. To reflect the direct health burden, the OOP/income ratio was constructed as the share of OOP medical payments in the previous year’s per capita income [29]. The effective reimbursement rate, as an indicator of the extent to how much of medical costs were covered, was the share of medical expenses paid by medical insurance in the respondents’ total medical expenses, including clinic and hospitalization expenditure [30].

The Andersen Behavioral Model of Health Service Use [31] provides a framework, with clear operational definitions and high predictability of behavior, to explore the access and utilization of medical services for vulnerable groups [32]. According to Andersen, predisposing factors comprised sex, age, education, marital status, occupation before 60 and standard household size; enabling characteristics included individuals’ pension and insurance cover and per capita income; and medical need-related characteristics comprised self-rated health, serious disease over the past two years and access to timely medical treatment [29,30,31].

From CLHLS, marital status was calculated as married and not married; family size was the number of people in the households; pre-retirement occupation was farmer and other occupation; income was all types of income, including pensions and wages. The variable serious disease over the past two years was changed into a dichotomous variable with ‘0’ and ‘1’—in which, ‘1’ represented that the respondent experienced severe illness one or more times. Self-rated economic status and self-rated health were multi-categorized variables in order—in which, ‘1’ to ‘5’ represented ‘very well’ to ‘very bad’.

### 2.3. Methods

To avoid survivor bias [33], we use a static three-equation seemingly unrelated regression (SUR) model framework for unbalanced panel data with latent individual heterogeneity [34,35]. The SUR model explains the variation of not just one dependent variable, as in the univariate multiple regression model, but the variation of a set of m dependent variables [36], estimating consistently better than the Ordinary Least Squares (OLS) does [37].

All SUR model versions we considered contained three equations comprising OOP payments, the effective reimbursement rate and OOP/income, with linear coefficients, which can be written compactly as G equations for observation (*i*; *t*): ***y***_it_ = X_it_***β*** + ***ε***_it_. For equation ***g***, ***x***_git_ is the repressor vector; ***ε***_git_ is the genuine disturbance and latent effect specific to individual ***i***, observation ***t***; ***β****_g_* is the coefficient vector (including the intercept term); and ***t*** is a sequence index, not a time index. Independent variables were selected based on the stepwise method, and ***β****_g_* was estimated by construction of a multistep algorithm using Generalized Least Squares and Maximum Likelihood procedures indicating the marginal effect of each independent variable. Three equations were built for OOP payments, the effective reimbursement rate and OOP/income percentage with independent variables. All analyses were performed using Stata SE 15.1 (Stata Corp; College Station, TX, USA).

## 3. Results

Table 1 summarizes the demographic and socioeconomic characteristics of rural elderly migrants from 2005 to 2014. The average age of the respondents averaged between 85.63 and 87.04 years old and the main occupation was farmer, accounting for approximately 85% of all occupations. Over time, the education level of the rural elderly increased, with the proportion of the elderly who have attended middle school and above increasing from 9.09% in 2005 to 15.60% in 2014. Further, elderly rural migrants who had severe illnesses over the last two years increased from 18.02% in 2005 to 26.19% in 2014. With improvement in access to health care facilities, the percentage of patients in China receiving immediate medical treatment was up to 95.46% by 2014. However, the average self-reported health status of elderly rural migrants declined slightly over the decade. In 2005, almost half of the elderly migrants reported that they were in good or very good health, which was down to 44% in 2014, and more respondents reported a “so so” health status subjectively. The descriptive statistics show that the income and medical insurance cover of elderly rural migrants with NCMS increased rapidly between 2005 and 2014. As shown in Table 1, NCMS coverage rose from 11.83% in 2005 to 87.33% in 2014 and elderly rural migrants who had some kind of income rose from 9.41% in 2005 to 27.17% in 2014. Access by elderly rural migrants to free public medical services, the UEBMI and other medical insurance remained relatively stable during this decade.

Table 2 shows that the medical expenses and the effective reimbursement rate for elderly rural migrants rose over the decade. The per capita income of rural migrant elders increased sharply from US$817.34 in 2005 to US$2118.77 in 2014 and medical expenses also more than doubled from an average of US$197.8 in 2005 to US$456.92 in 2014. OOP payments averaged US$176.15 in 2005, accounting for 22.92% of total medial costs, rising to a peak of US$277.95 in 2011 (15.61% of total medical costs) before falling to US$202.09 (11.22% of total medical costs) in 2014. The effective reimbursement rate rose significantly from 4.53% in 2005 to 36.44% in 2014, with the OOP/income percentage of medical expenses falling by half.

The SUR model coefficients in Table 3 represent the marginal effect of the influencing factors on OOP payments, OOP/income percentage and the effective reimbursement rate. A variety of demographic factors, socioeconomic factors and medical insurance type significantly impacted OOP expenses and the effective reimbursement rate. The social demographic characteristics that significantly affected OOP medical expenses and OOP/income proportion were similar. Age, sex, occupation, marital status and education level of the respondents had significant effects on OOP medical expenses and OOP/income proportion, but no significant impact on the effective reimbursement rate. The variables on the demand and utilization of medical services, poor self-rated health, adequate health care and severe disease in the last two years brought significant marginal effects to the proportion of OOP/income. When elderly rural migrants suffered from one or more severe diseases in the past two years, their marginal OOP medical expenses increased by $2.864, the reimbursement rate increased by 6.3% and the share of OOP medical expenses in per capita income was raised by 21.6%. Higher per capita income implied more OOP medical expenditure and lower OOP/income percentage.

The NCMS had an insignificant decline of $1.060 in OOP medical payments over the decade and the UEBMI and free public health care had no significant influence on OOP expenditures. The NCMS significantly increased the actual reimbursement rate by 12.4% and OOP/income proportion by 7.5%. Public health care and the UEBMI effectively promoted the reimbursement rate but had no significant impact on the OOP/income and OOP expenditures.

## 4. Discussion

Urbanization in China meant increasing numbers of elderly people left their rural homes and migrated to cities [4]. The demographics of aging raise global economic, political, social, and public health concerns [38,39,40]. China’s situation is particularly challenging because it is projected to have the largest number of citizens aged 80 and above by 2050 [39]. While some research exists on the general health of the elderly population in China, this study contributes specific findings on elderly rural migrants in urban cities, validates the magnitude of the medical economic burden, and raises consciousness about the ramifications of national health insurance. Elderly rural migrants did not enjoy equal access to health welfare comparable to elderly non-migrants, with medical insurance as one illustrative example. The vast majority, roughly 85%, of elderly rural migrants only had the NCMS as their medical insurance, and policy conditions presented obstacles to accessing health care in urban places where elderly rural migrants were now resident. The NCMS was reformed after 2009, but the direct medical financial burden on elderly rural migrants of OOP medical payments was not attenuated by the NCMS or other medical insurance. However, the proportion of OOP/income was reduced due to NCMS reimbursement rate changes, rises in per capita income and marital status.

Migration was a double-edged sword for elderly rural migrants, incurring medical expenses. On one hand, direct medical expenses increased year by year, and medical insurance did not play an effective protective role, with OOP payments increasing. The welfare effects of the NCMS were offset by the excessive growth of medical expenses [41], which is reflected in the NCMS not reducing OOP expenses. On the other hand, the NCMS significantly reduced the relative medical and economic burden, measured by the proportion of reimbursements and the proportion of OOP expenditures. There may be three reasons for this outcome. First, during the period 2005–2014, the total proportion of all kinds of income earned by elderly migrant people increased from 9.41% to 27.17%, which brought about the improvement of their ability to pay for health services. Second, the majority of elderly rural migrants lived with their children [42] in urban locations, leading to rising per capita incomes, which enhanced the ability of the elderly rural migrants to financially withstand health service risks and costs. Third, elder migrants, worried about the high medical costs and the financial burden on themselves and their children, went without medical care [15]. The discrimination against migrants in national insurance policies constrained migrants’ access and utilization of health care, depriving them of part of their migration dividend of moving to urban areas [43,44].

This study found that poor self-rated health and severe illness in the past two years increased the direct medical economic burden and medical expenditure of the elderly migrants, which partly reflects the increased utilization of medical services in this population. Previous studies found that the self-rated health status of mobile elderly is negatively correlated with their utilization of health services [45]. Our study also found that elderly migrants with spouses and with higher per capita incomes experienced a significant increase in their OOP medical expenditure. Social support, reflected by living with a spouse, and higher family income encouraged medical treatment, which had a crucial impact on the medical expenditure of elderly migrants. The increase in the per capita income of the rural elderly migrants, partly from the financial support of their children and partly from an increased proportion of households with an income, led to a significant reduction of their direct medical financial burden in the OOP/income percentage, but may also mislead policy makers to ignore the financial vulnerability of the elderly migrant population and the severity of their disease burden.

Reimbursement rates for elderly migrants increased significantly due to the NCMS—a pattern also reflected in other medical insurance schemes, such as the UEBMI. However, the increase in the effective reimbursement rate did not bring a decrease in OOP medical expenses, which indicates that NCMS medical insurance compensation did not offset the increase in medical expenses. Elderly rural migrants’ rapidly growing medical costs, and rising OOP payments, can be explained by several factors. First, after the rural elderly migrants moved to the city, their access to medical services improved and diseases that were difficult to diagnose and went untreated in the past were treated. The proportion of respondents with a positive answer to ‘access to timely medical treatment’ and ‘serious disease over the past two years’ increased over time, supporting our inference on enhanced access to health services. Second, due to the narrow reimbursement scope of the NCMS that the majority of elderly rural migrants have, new medical technologies and drugs may not be included in the existing reimbursement guidelines [46], and accessing these technologies and new drugs, especially in urban hospitals, increased medical expenses and OOP payments. Finally, the reimbursement scope and upper limits on individual NCMS accounts were more limited than those of UEBMI and URBMI holders because of lower funding levels [47]. Since NCMS reimbursement policy upper limits for outpatient medical services were usually quite low [47], the NCMS failed to compensate adequately for the outpatient medical expenses of the elderly rural migrants. 

As international and internal migration grow in scope and complexity, migrants are at risk of exclusion from universal health coverage [48]. For example, in Ghana, nearly half of the floating population did not have medical insurance, and only one-tenth of the immigrants carried health insurance cards, leading to unsatisfaction in medical service utilization [48]. Policy makers need to promote a two-pronged strategy that involves controlling the excessive growth of urban medical expenses and solving the problem of reimbursement for medical treatment in different places, so that elderly rural migrants can fully enjoy the welfare benefits of urbanization. The 2009 health reforms, including changes to the national health insurance schemes, did not fully address elderly rural migrants’ rising OOP medical expenditures. Compared with other basic medical insurance systems, the NCMS urgently needs to further reform the payment mode in order to effectively contain medical cost growth and rising personal health costs, limiting medical debt and excessive medical bills. Since 2014, the Chinese government has gradually released the nationwide networking of basic medical insurance and the cross-provincial direct reimbursement scheme. By the end of September 2017, all provinces and coordinated areas in the country had some access to the national off-site internet medical settlement system covering participants enrolled in basic medical insurance schemes, including the NCMS. The 2017 nationwide reform of the NCMS settlement policy, which will reimburse medical expenses of rural migrants, including retirees, who had settled in non-hukou urban places, should be analyzed once data are available. 

Compared with previous studies on Chinese elderly rural migrants, this study has a larger geographical span and covers approximately 85% of the population in China, and so it is more representative of the macro trends in elderly migration. Moreover, this study spans 2005–2014, reflecting the impact of the 2009 reforms on the development of China’s medical insurance for the rural elderly. Finally, and most importantly, our SUR model addresses the survivor bias problem in other studies, generating more accurate estimation results, providing an empirical reference point for medical insurance policy makers. 

There were some limitations in this study. Some respondents lacked hospitalization reimbursement in 2014, which may lead to the underestimation of OOP payments. CLHLS questionnaires measure household per capita household income (2005) or the total household income (2008, 2011, 2014), which provides household averages rather than representing the income of the elderly migrants themselves. This would, of course, mean our estimates were lower bound.

## 5. Conclusions

The self-reported rise in poorer health status, growth in per capita income and access to urban health services meant that elderly rural migrants bought more medical services. By 2014, NCMS coverage of elderly rural migrants reached 87.33% and the effective reimbursement rate had increased by 12.4% and OOP/income proportion had increased by 7.5%. However, the 2009–2014 reforms to NCMS coverage and reimbursement did not fully address elderly rural migrants’ rising OOP medical expenditures. Controlling the growth rate of medical expenses is equally important with non-hukou reimbursements for supporting elderly rural migrants insured by the NCMS. Further post-2104 policy changes to improve non-hukou elderly rural migrants’ medical cost reimbursements were a move in the right direction, but data are not available to assess this important change. Controlling medical costs and ongoing health and social welfare reforms to NCMS reimbursements will further reduce the direct medical economic burden on elderly rural migrants.

## Figures and Tables

**Table 1:** Demographic Characteristics of the Samples.

Variables	*n* (%)			
	2005	2008	2011	2014
	(N = 8658)	(N = 10198)	(N = 5132)	(N = 3967)
Age	86.08 ± 11.69	87.04 ± 11.84	85.85 ± 11.68	85.63 ± 10.96
Family Size	3.53 ± 2.07	3.8 ± 1.88	3.73 ± 2.08	3.32 ± 1.9
Sex				
Male	3643 (42.08)	4272 (41.89)	2268 (44.19)	1814 (45.73)
Female	5015 (57.92)	5926 (58.11)	2864 (55.81)	2153 (54.27)
Occupation Category				
Farmer	7061 (83.42)	8556 (85.73)	3696 (85.58)	2840 (84.20)
Other	1403 (16.58)	1424 (14.27)	623 (14.42)	533 (15.80)
Education Level				
No Schooling	5925 (68.43)	6953 (68.18)	3280 (63.91)	2473 (62.34)
Primary School	1946 (22.48)	2096 (20.55)	1139 (22.19)	875 (22.06)
Middle School and Above	787 (9.09)	1149 (11.27)	713 (13.89)	619 (15.60)
Marital Status				
Not Married	2752 (31.79)	3237 (31.74)	1929 (38.00)	1508 (39.69)
Married	5906 (68.21)	6961 (68.26)	3147 (62.00)	2291 (60.31)
Self-Rated Health				
Very Good	778 (9.78)	921 (10.39)	413 (8.76)	238 (6.63)
Good	3137 (39.44)	3366 (37.98)	1671 (35.44)	1354 (37.72)
So So	2666 (33.52)	3007 (33.93)	1800 (38.18)	1457 (40.58)
Bad	1233 (15.5)	1437 (16.21)	772 (16.37)	488 (13.59)
Very Bad	140 (1.76)	132 (1.49)	59 (1.25)	53 (1.48)
Access to Timely Medical Treatment				
Yes	7377 (85.2)	9172 (89.94)	4712 (92.96)	3649 (95.47)
No	1281 (14.8)	1026 (10.06)	357 (7.04)	173 (4.53)
Serious Disease Over the Past Two Years				
Never	7098 (81.98)	8630 (84.62)	4091 (79.72)	2928 (73.81)
Once or More	1560 (18.02)	1568 (15.38)	1041 (20.28)	1039 (26.19)
Income				
Yes	815 (9.41)	1259 (12.35)	1224 (23.85)	1078 (27.17)
No	7843 (90.59)	8939 (87.65)	3908 (76.15)	2889 (72.83)
Free Public Medical Services				
Yes	181 (2.09)	212 (2.08)	75 (1.51)	50 (1.41)
No	8476 (97.91)	9986 (97.92)	4907 (98.49)	3497 (98.59)
Urban Employee Basic Medical Insurance (UEBMI)				
Yes	253 (2.92)	670 (6.57)	121 (2.43)	117 (3.29)
No	8404 (97.08)	9528 (93.43)	4862 (97.57)	3437 (96.71)
New Cooperative Medical Scheme (NCMS)				
Yes	1024 (11.83)	6758 (66.27)	4293 (84.84)	3280 (87.33)
No	7633 (88.17)	3440 (33.73)	767 (15.16)	476 (12.67)
Other Medical Insurance				
Yes	267 (3.08)	430 (4.22)	148 (2.88)	114 (2.87)
No	8391 (96.92)	9768 (95.78)	4984 (97.12)	3853 (97.13)

**Table 2:** Medical Expenses, Out-of-Pocket (OOP) Expenses and the Effective Reimbursement Ratio.

Variables	Mean ± SD			
	2005	2008	2011	2014
Medical Expenses ($)	197.8 ± 604.96	247.18 ± 791.45	415.25 ± 1370.21	456.92 ± 1570.63
OOP Payment ($)	176.15 ± 488.87	212.22 ± 681.86	277.95 ± 815.74	202.09 ± 879.17
Per Capita Income ($)	817.34 ± 1517.26	1487.81 ± 1582.60	1998.45 ± 2110.42	2118.77 ± 2263.04
OOP/Income (%)	22.92 ± 32.58	14.98 ± 26.78	15.61 ± 29.26	11.22 ± 24.79
Effective Reimbursement Rate (%)	4.53 ± 18.06	6.82 ± 19.35	22.06 ± 31.53	36.44 ± 36.92

**Table 3:** Influence Factors of OOP payment, OOP/Income and NCMS Reimbursement.

Independent Variables	Regression Coefficient (Significance)		
	OOP Payment (Logarithm)	Effective Reimbursement Rate	OOP/Income Percentage
**Predisposing Characteristics**			
Age	0.012 **	0.000	0.004 **
Sex	0.133 **	0.002	0.036 **
Marital Status	−0.148 **	−0.015	−0.057 **
Wage	−0.013	0.113 **	−0.014
Farmer	0.105 **	0.014	0.050 **
Standard Family Size ^1^	0.019	−0.005	0.003
Education	0.083 **	0.004	0.026 **
**Medical Need Characteristics**			
Self-Rated Health	0.153 **	−0.002	0.055 **
Access To Timely Medical Treatment	0.090	0.008	0.055
Serious Disease Over the Past Two Years	0.457 **	0.063 **	0.210 **
**Enabling Characteristics**			
Per Capita Income	0.052 **	0.005	−0.090 **
UEBMI	0.032	0.135 **	0.006
NCMS	−0.025	0.124 **	−0.075 **
Public Free Health Care	−0.202	0.197 **	0.000
Other Medical Insurance	−0.125	0.022	−0.004

Note: “**” means *p* < 0.0. ^1^ Standardized family size is family size^0.56.

## References

[1] Meng Q., Fang H., Liu X., Yuan B., Xu J. (2015). Consolidating the social health insurance schemes in China: Towards an equitable and efficient health system. Lancet.

[2] Mou J., Griffiths S.M., Fong H., Dawes M.G. (2013). Health of China’s rural-urban migrants and their families: A review of literature from 2000 to 2012. Brit. Med. Bull..

[3] Chen Y. (2011). Rural migrants in urban China: Characteristics and challenges to public policy. Local Econ. J. Local Econ. Policy Unit.

[4] China NHCO (2019). China Floating Population Development Report 2018.

[5] Singh N.S., Bass J., Sumbadze N., Rebok G., Perrin P., Paichadze N., Robinson W.C. (2018). Identifying mental health problems and Idioms of distress among older adult internally displaced persons in Georgia. Soc. Sci. Med..

[6] Wang B., Yang H., Ning J., Fan Z., Herry L., Qiu J., Zhou J. (2011). Investigation on physical and mental health of immigrant and non-immigrant elderly in three gorges reservoir area. Chongqing Med..

[7] Woo J., Kwok T., Sze F., Yuan H.J. (2002). Ageing in China: Health and social consequences and responses. Int. J. Epidemiol..

[8] Dai T., Hu H.P., Na X., Li Y.Z., Wan Y.L., Xie L.Q. (2016). Effects of new rural cooperative medical scheme on medical service utilization and medical expense control of inpatients: A 3-year empirical study of Hainan province in China. Chin. Med. J..

[9] Guo J., Guan L., Fang L., Liu C., Fu M., He H., Wang X. (2017). Depression among Chinese older adults: A perspective from Hukou and health inequities. J. Affect. Disord..

[10] Chen W., Zhang Q., Renzaho A.M.N., Zhou F., Zhang H., Ling L. (2017). Social health insurance coverage and financial protection among rural-to-urban internal migrants in China: Evidence from a nationally representative cross-sectional study. BMJ Glob. Health.

[11] Giles J., Wang D., Park A. (2013). Expanding social insurance coverage in urban China. Res. Labor Econ..

[12] Yongyi W., Wei W., Fei Y. (2019). Health Services Utilization and Influencing Factors in Elderly Migrants in Shanghai: A Mixed-method Study. Chin. Gen. Med..

[13] Liu D., Tsegai D., Litaker D., von Braun J. (2015). Under regional characteristics of rural China: A clearer view on the performance of the New Rural Cooperative Medical Scheme. Int. J. Health Econ. Manag..

[14] Nan Jia J.M. (2015). Research on the locking effect of non-portable medical insurance on rural labor mobility. Manag. World.

[15] Yongyi W., Wei W., Fei Y. (2017). Qualitative research on utilization of health services for floating elderly in Shanghai. Med. Soc..

[16] Cheng L., Liu H., Zhang Y., Shen K., Zeng Y. (2015). The impact of health insurance on health outcomes and spending of the elderly: Evidence from China’s new cooperative medical scheme. Health Econ..

[17] Liang X., Guo H., Jin C., Peng X., Zhang X. (2012). The effect of new cooperative medical scheme on health outcomes and alleviating catastrophic health expenditure in China: A systematic review. PLoS ONE.

[18] Chen J., Yu H., Dong H. (2016). Effect of the new rural cooperative medical system on farmers’ medical service needs and utilization in Ningbo, China. BMC Health Serv. Res..

[19] Xie F., Jiang X., Yuan F., Chen X., Yuan Z., Lu Y. (2018). Impact of the new cooperative medical scheme on the rural residents’ hospitalization medical expenses: A five-year survey study for the Jiangxi Province in China. Int. J. Environ. Res Public Health.

[20] Hou Z., Van de Poel E., Van Doorslaer E., Yu B., Meng Q. (2014). Effects of NCMS on access to care and financial protection in CHINA. Health Econ..

[21] Zhao J., Su Y., Mao Y., Chen A., Zhou X., Zhou W., Zhu Q. (2019). Intended place of residence in old age of internal migrants aged 15–64 years: A citywide cross-sectional study in Shanghai, China. BMJ Open.

[22] Peng B., Ling L. (2019). Association between rural-to-urban migrants’ social medical insurance, social integration and their medical return in China: A nationally representative cross--sectional data analysis. BMC Public Health.

[23] Han J., Meng Y. (2019). Institutional differences and geographical disparity: The impact of medical insurance on the equity of health services utilization by the floating elderly population-evidence from China. Int. J. Equity Health.

[24] Zeng Y., Li J., Yuan Z., Fang Y. (2019). The effect of China’s new cooperative medical scheme on health expenditures among the rural elderly. Int. J. Equity Health.

[25] Smith J.P. (1999). Healthy bodies and thick wallets: The dual relation between health and economic status. J. Econ. Perspect..

[26] Ettner S.L. (1996). New evidence on the relationship between income and health. J. Health Econ..

[27] Van Minh H., Kim Phuong N.T., Saksena P., James C.D., Xu K. (2013). Financial burden of household out-of-pocket health expenditure in Viet Nam: Findings from the National Living Standard Survey 2002–2010. Soc. Sci. Med..

[28] Shen P., Zhang J.K. (2010). Research on social discount rate in cost-benefit analysis of public health projects. Chin. Health Econ..

[29] Ukwaja K.N., Alobu I., Abimbola S., Hopewell P.C. (2013). Household catastrophic payments for tuberculosis care in Nigeria: Incidence, determinants, and policy implications for universal health coverage. Infect. Dis. Poverty.

[30] Pan Y., Chen S., Chen M., Zhang P., Long Q., Xiang L., Lucas H. (2016). Disparity in reimbursement for tuberculosis care among different health insurance schemes: Evidence from three counties in central China. Infect. Dis. Poverty.

[31] Andersen R.M. (1995). Revisiting the behavioral model and access to medical care: Does it matter?. J. Health Soc. Behav..

[32] Lee S., Choi S. (2014). Disparities in access to health care among non-citizens in the United States. Health Sociol. Rev..

[33] Han W., Shibusawa T. (2015). Trajectory of physical health, cognitive status, and psychological well-being among Chinese elderly. Arch. Gerontol. Geriat..

[34] Biørn E. (2014). Estimating SUR system with random coefficients: The unbalanced panel data case. Empir. Econ..

[35] Biørn E. (2004). Regression systems for unbalanced panel data: A stepwise maximum likelihood procedure. J. Econom..

[36] Zellner A. (1962). An efficient method of estimating seemingly unrelated regressions and tests for aggregation bias. Econometrica.

[37] Keshavarzi S., Ayatollahi S.M.T., Zare N. (2013). Quality of life of childbearing age women and its associated factors: An application of seemingly unrelated regression (SUR) models. Qual. Life Res..

[38] Cheng Y., Gao S., Li S., Zhang Y., Rosenberg M. (2019). Understanding the spatial disparities and vulnerability of population aging in China. Asia Pac. Policy Stud..

[39] Su S.W., Wang D. (2019). Health-related quality of life and related factors among elderly persons under different aged care models in Guangzhou, China: A cross-sectional study. Qual. Life Res..

[40] Yang Z., Huo A. (2019). Optimization of rural mutual assistance pension mode under the background of aging population. Proceedings of the 3rd International Conference on Economics and Management, Education, Humanities and Social Sciences (EMEHSS 2019).

[41] Liu X., Sun X., Zhao Y., Meng Q. (2016). Financial protection of rural health insurance for patients with hypertension and diabetes: Repeated cross-sectional surveys in rural China. BMC Health Serv. Res..

[42] Lin X., Bryant C., Boldero J., Dow B. (2015). Older Chinese immigrants’ relationships with their children: A literature review from a solidarity-conflict perspective. Gerontologist.

[43] Wang Q. (2017). Health of the elderly migration population in China: Benefit from individual and local socioeconomic status?. Int. J. Environ. Res. Public Health.

[44] Jutz R. (2015). The role of income inequality and social policies on income-related health inequalities in Europe. Int. J. Equity Health.

[45] Bei W., Man G., Chi I., Plassman B.L. (2011). Social network and health: A comparison of Chinese older adults in Shanghai and elderly immigrants in Boston. Int. J. Soc. Welf..

[46] Hu J., Mossialos E. (2016). Pharmaceutical pricing and reimbursement in China: When the whole is less than the sum of its parts. Health Policy.

[47] Zhang X., Wu Q., Shao Y., Fu W., Liu G., Coyte P.C. (2015). Socioeconomic inequities in health care utilization in China. Asia Pac. J. Public Health.

[48] Lattof S.R. (2018). Health insurance and care-seeking behaviours of female migrants in Accra, Ghana. Health Policy Plan..

